# Classical Swine Fever: A Truly Classical Swine Disease

**DOI:** 10.3390/pathogens9090745

**Published:** 2020-09-10

**Authors:** Fun-In Wang, Chia-Yi Chang

**Affiliations:** 1 School of Veterinary Medicine, National Taiwan University, No. 1, Section 4, Roosevelt Road, Taipei 10617, Taiwan; 2 OIE Reference Expert for CSF, Animal Health Research Institute, Council of Agriculture, Executive Yuan, 376 Chung-Cheng Road, Tansui, New Taipei City 25158, Taiwan; cychang@nvri.org.gov.tw

Recent reemergence of classical swine fever (CSF) in previous CSF-free areas reminds the veterinary community of this old disease. At this difficult period, ***Pathogens*** has the timely honor to present a ***Special Issue on Classical Swine Fever*** collecting 14 publications. Readers can find that perhaps few swine diseases have such a comparable ancient history as CSF [1], and few have such a versatile capability in affecting so many body systems [1] via horizontal [2,3] as well as vertical [4,5] transmissions. And few have so many clinical forms expressed, ranging from acute to chronic, atypical to congenital, etc. [1,4].

The CSF world has been quiet for almost 20 years [6]. It was until the year 2018, when a reemergence from previously CSF-free Japan [2,3] raised our attention. The reemergence was largely attributed to the virus hidden in wild boar populations and transmitted, by direct or indirect contact, to neighboring domestic herds. The spread of CSF followed the migration paths of wild boars. The same concern also goes to wild boar populations residing in the demilitarized zone of Korea [7]. The spatial distance between each CSF notification was about 23 km, the widest radius of outbreak cluster was 20 km [2], and each outbreak cluster lasted 98–124 days [2]. These data provide a scientific basis on how far and how long the control measure should be imposed. Monitoring the antigen and antibody in wild boars will provide warning for neighboring domestic pigs in those particular settings [3,7].

The threat from CSF remains. The continual application of newer technologies, such as next generation sequencing coupled with meta-analysis, point-of-care diagnosis [8,9], as well as the continual development of more specific and sensitive diagnostics [8,10] and vaccine [9] testify for its potential threat. Even when a country has been CSF-free, monitoring for the risk of reemergence is necessary. Viremia is a key step in CSF pathogenesis and serum remains a preferred testing sample for detecting the antibody, antigen, or nucleic acid. Thus, serum itself may become a vehicle of disease spread [11], not only for CSF virus (CSFV) but also for others such as porcine reproductive and respiratory syndrome virus (PRRSV) [12], so that inactivation of viruses while not disturbing antibody detection is key to prevent such risk [11].

Vaccination has been practiced for years with success. The question is: is the currently used modified live virus (MLV) vaccine really as safe as we thought [5,13]? The MLVs were developed years ago and their degrees of attenuation were characterized by traditional methods. MLVs are favored for its induction of cell-mediated immunity, which is not possible by killed or subunit vaccines [14]. A recent outbreak, which occurred in a previously CSF-free island carrying MLV vaccination, led to a later study showing that the employed MLV can cause viremia and cross the placenta to piglets [5,13]. This reminds us of the need to recharacterize the MLV using more recent technologies, such as reverse-transcription polymerase chain reaction, to meet the OIE (The World Organization for Animal Health) standards [15]. MLV has the further disadvantage of overloading the immune system, when multiple infections with various viral and bacterial pathogens, such as PRRSV, occur regularly in the field [12]. Thus “more is not necessarily better” for a busy immune system, wherein killed or subunit CSF vaccines are suitable [12].

Colleagues in the CSF world are familiar with a recent “virus shift” from genotype 3 to genotype 2 in the field. We now know that genotype 2.1 has an in vivo replication advantage of 1.5–3 log over that of genotype 3.4, which partially explains the virus shift observed in the field [16], although other mechanisms are certainly involved. The detection of “virus shift” is benefited by the phylogenetic analysis, which is useful to trace the origin of the outbreak of the virus. It is found that most CSFVs circulating in North Vietnam belong to subgenotype 2.1c [17], similar to those strains circulating in the geographically proximal Southern China. This ruled out a possible outbreak derived from an unsafe MLV vaccine [5,13] applied.

The transplacental transmission and congenital form of CSF is always a concern, since it is a potential source of persistent infection in the herd. Indeed, experimentally infecting sows at mid-gestation showed newborn viremic piglets launching CD8^+^-T cell and interferon (IFN) -alpha responses to CSFV [4], and fast and solid immunity for sows is required for prevention of congenital viral persistence. The versatility of CSFV in causing disease culminates in its ability to manipulate several biological processes, namely apoptosis, autophagy, and mitophagy, and pyroptosis for its own advantage [18]. These pathogeneses cannot be detected by routine diagnostic procedures [8,15], and further molecular characterization is required.

The CSF is a truly classical swine disease that will continue to pose a threat to pig production. Our fight against CSF is far from over, and it deserves our continual attention.
